# Pharmacogenomics in Pediatric Oncology: Review of Gene—Drug Associations for Clinical Use †

**DOI:** 10.3390/ijms17091502

**Published:** 2016-09-08

**Authors:** Vid Mlakar, Patricia Huezo-Diaz Curtis, Chakradhara Rao Satyanarayana Uppugunduri, Maja Krajinovic, Marc Ansari

**Affiliations:** 1 Cansearch Research Laboratory, Geneva University Medical School, Avenue de la Roseraie 64, 1205 Geneva, Switzerland; patricia.curtis@unige.ch (P.H.-D.C.); chakradhara.uppugunduri@unige.ch (C.R.S.U.); marc.ansari@hcuge.ch (M.A.); 2 Charles-Bruneau Cancer Center, Centre hospitalier universitaire Sainte-Justine, 4515 Rue de Rouen, Montreal, QC H1V 1H1, Canada; maja.krajinovic@umontreal.ca; 3 Department of Pediatrics, University of Montreal, 2900 Boulevard Edouard-Montpetit, Montreal, QC H3T 1J4, Canada; 4 Department of Pharmacology, Faculty of Medicine, University of Montreal, 2900 Boulevard Edouard-Montpetit, Montreal, QC H3T 1J4, Canada; 5 Pediatric Department, Onco-Hematology Unit, Geneva University Hospital, Rue Willy-Donzé 6, 1205 Geneva, Switzerland

**Keywords:** pediatrics, pharmacogenomics, PharmGKB, thiopurine, cisplatin, methotrexate, cyclophosphamide, irinotecan

## Abstract

During the 3rd congress of the European Society of Pharmacogenomics and Personalised Therapy (ESPT) in Budapest in 2015, a preliminary meeting was held aimed at establishing a pediatric individualized treatment in oncology and hematology committees. The main purpose was to facilitate the transfer and harmonization of pharmacogenetic testing from research into clinics, to bring together basic and translational research and to educate health professionals throughout Europe. The objective of this review was to provide the attendees of the meeting as well as the larger scientific community an insight into the compiled evidence regarding current pharmacogenomics knowledge in pediatric oncology. This preliminary evaluation will help steer the committee’s work and should give the reader an idea at which stage researchers and clinicians are, in terms of personalizing medicine for children with cancer. From the evidence presented here, future recommendations to achieve this goal will also be suggested.

## 1. Introduction

Twenty percent of pediatric cancer patients do not respond to standard therapy [1] and 22% of all hospital admissions in this population is due to adverse drug reactions (ADRs) [2]. The therapeutic agents used in cancer chemotherapy are often administered at high doses [3], which due to inter-patient variability and narrow therapeutic ranges result in a spectrum of outcomes from severe toxicities to underexposure. Part of this variability can be attributed to heritable genetic variations affecting the drug pharmacokinetics and pharmacodynamics. The study of the relationship between genetics and drug function is most commonly known as pharmacogenetics or pharmacogenomics. Pharmacogenomics has the potential to improve the drug safety and efficacy and is recognized as a valid approach to personalize treatment [4,5].

Patients respond differently to medication due to their constitutive genetic variations but also due to mutations or epigenetic signatures acquired during the process of neogenesis or treatment. The intention of this review is to focus on germline variations that might affect treatment efficacy and toxicity. For a detailed revision of acquired mechanisms of cancer resistance, we recommend reviews by Holohan et al. and Longley et al. [6,7].

The establishment of a pediatric individualized treatment in oncology and hematology committee was proposed during the 3rd congress of The European Society of Pharmacogenomics and Personalised Therapy (ESPT) (Budapest, Hungary, 7–9 October 2015) with the aim to facilitate the transfer and harmonization of pharmacogenetics testing in children, bring together basic and translational research, educate health professionals throughout Europe and establish partnerships for industry and regulatory bodies. A summary of our current knowledge in pharmacogenomics is essential to achieve these aims.

## 2. The Role of Ontogeny in Pharmacogenomics

Pharmacogenomics in children, unlike adults, must be viewed in the context of body development in addition to the physiological changes due to illness. As the child grows into an adult, changes occur in the body composition. For example, premature neonates of approximately 1.5 kg have 3% of body fat, which raises to 12% by the 40th week of gestation; and then up to 25% by the 4th month of age. Similarly, protein mass increases from 20% at birth to 50% in the adult [8]. These changes must be considered when analyzing the differences in pharmacokinetic data in relation to the genotypes. Another issue of importance for clinical utility of pharmacogenetic tests in children is ontogeny of drug metabolizing enzymes (DMEs) (Phase 1 and 2), transporters, and target proteins. A prime example is development of drug metabolism capability associated with expression of P450 cytochromes (CYP) [9]. Enzymes of the CYP3A family have been shown to substantially change their activity from foetus to adulthood. The family has four members in humans: 3A4, 3A5, 3A7 and 3A43. CYP3A4 is the most abundantly expressed CYP in liver and small intestine accounting for 30%–40% of CYP proteins. CYP3A4 has extremely low activity at birth, reaching approximately 30%–40% of adult activity by the first month and full adult activity by the 6th month; exceeding adult activity (120%) between 1 and 4 years of age, decreasing to adult levels after puberty [10]. CYP3A5 is 83% homologous with CYP3A4 and is expressed in kidney and in liver but at lower levels. CYP3A5 with CYP2B6 are the only two CYPs along with a phase II enzyme *N*-acetyl transferase with no variable expression from childhood to adulthood suggesting equal importance of their genetic variants at every developmental stage. CYP3A7 is 90% homologous with CYP3A4 and is mostly expressed in embryonic, foetal and new-born liver [11]. Similar variability in expression during development is noted for CYP2D6. It was demonstrated that foetuses less than 30 weeks old show less than 5% of CYP2D6 activity in comparison to adults. After birth, the activity gradually increases such that between day 7 and 28 the activity is 30% and between 4 weeks and 5 years the activity is 70% to that of adults [12]. In addition CYP expression and activity can be affected by common health problems such as non-alcoholic fatty liver disease and neonatal diabetes.

Much like DMEs, ontogeny of drug transporters and drug targets is also important when it comes to the evaluation and implementation of pharmacogenetics. Multidrug resistance protein 1 (MDR1 or P-gp) and ATP binding cassette (ABC) G2 are expressed early in childhood, whereas other transporters like organic anion transporter (OATP1B1) and multidrug resistant protein 2 (MRP2) exhibit delayed maturation and reduced expression levels during the first months of childhood compared to adults [13].

The above described cases are just a few examples of ontogeny contribution to gene expression differences. A recent publication [14] showed that up to 688 genes are differentially expressed during childhood development only in lymphoblastic cells. Using gene expression profiling of lymphoblast cells, researchers were able to distinguish three particular groups: pre-pubertal (under 6), pubertal (from 6 to 17) and early adulthood group (older than 17) [5,14] suggesting a set of developmental genes which are expressed in an independent manner [5,15].

The importance of ontogeny is reflected by different responses to the same drug by children and adults. Higher susceptibility to ototoxicity with cisplatin treatment [16,17], effects on neurological development linked to methotrexate [18], higher clearance of tacrolimus or unresponsiveness to codeine in infants [19] are but a few such examples. Lastly, certain diseases such as acute lymphoblastic leukemia (ALL), neuroblastoma (NB) and osteosarcoma (OS) appear predominantly in children and must be therefore linked to ontogeny, providing additional support that pediatric pharmacogenomics should be considered as a distinct field. Scarcity of information and consensus on ontogeny is still one of the major limitations for a clear understanding of utility of genetic variants [20].

## 3. Methods

**Figure 1:**
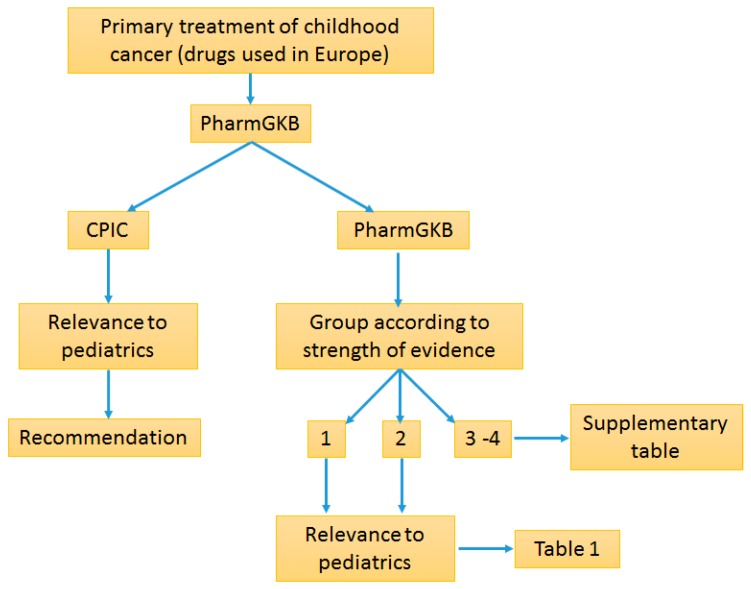
Methodology flow chart.

The initial step prior to assessing gene–drug relationships was to list all the current major drugs in Europe used to treat the following conditions in children: leukemias, lymphomas, brain tumors and solid tumors (see Supplementary Materials Table S1) [21]. In order to simplify the search, only primary drugs were included and supportive treatment such as prophylaxis drugs or co-medications were excluded. The second step was to identify whether these drugs already had established gene relationships on The Pharmacogenomics Knowledgebase (PharmGKB, https://www.pharmgkb.org/) web-based database or are incorporated in clinical guidelines of Clinical Pharmacogenetics Implementation Consortium (CPIC) (Figure 1). PharmGKB is an online comprehensive resource that curates knowledge on the impact of genetic variations on drug responses for clinicians and researchers. It is dedicated to systematically extract and evaluate evidence for gene/drug associations from scientific databases. PharmGKB collaborates closely with CPIC whose task is to prepare clinical guidelines for gene/drug pairs that satisfy the highest standards of evidence and has clinical significance [22].

Flowchart of methodology used to extract information on pediatric oncology pharmacogenomics. First, drugs used in Europe were defined (Supplementary Materials Table S1). CPIC and PharmGKB was used to search for guidelines and drug/gene associations and strength of evidence. Genes were grouped according to the evidence in three groups (Supplementary Materials Table S1). Lastly, studies published in children and pediatric sections of CPIC were selected and reviewed (Table 1).

PharmGKB curators rate gene/drug associations into four broad groups based on the strength (1 strongest to 4 weakest) of evidence for association. In short, level 1 includes gene/drug associations that show significant *p* values in more than one cohort and preferably with strong effect size; Level 2 includes associations that were replicated but other studies that do not show significance may be present and/or association shows small effect size; Level 3 is based on single studies showing significant association but the evidence has not been replicated; Level 4 is based on case reports, in vitro, molecular or other functional assays only (for a detailed explanation please refer to the original manuscript) [22]. The system used in this review adapted the PharmGKB approach with a few modifications in that group 3 and 4 were combined into a single group of low evidence gene/drug associations having low probability of entering clinical level at the time and group 1 and 2 remained separate (see Table S1). Next, we extracted publications on drug/gene associations with high and moderate evidence (Groups 1 and 2) for drugs most commonly used in pediatric oncology medicine. Each publication was checked for patient population used. Publications in pediatrics were extracted and used for review. Lower evidence drug/gene pairs (Levels 3 and 4) were discussed in case it contributed to better understanding of higher level drug/gene associations.

## 4. Results

### 4.1. Drugs with Strong Pharmacogenetics Evidence

From the initial screen, we were able to identify the following drug/gene pairs with strong pharmacogenetic evidence (Group 1): thiopurines/thiopurine *S*-methyltransferases (*TPMT*), thiopurines/nudix hydrolase 15 (*NUDT15*) and cisplatin/Xeroderma Pigmentosum, Complementation Group C (*XPC*) (Table 1) with thiopurines/TPMT already having published guidelines by CPIC [255,256].

#### 4.1.1. Thiopurines/Thiopurine *S*-Methyltransferases (*TPMT*) Pair

**Figure 2:**
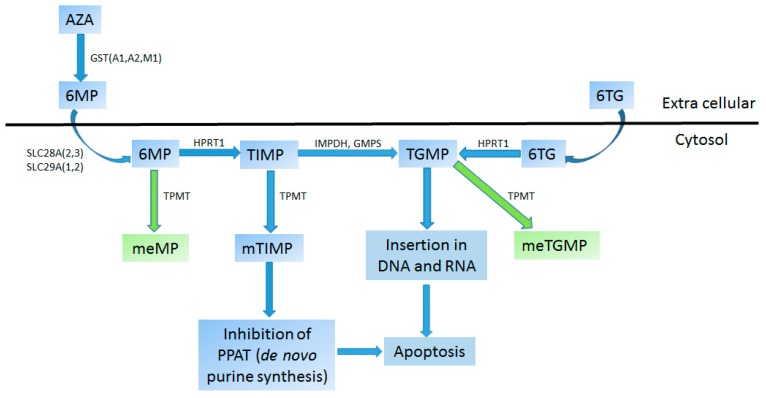
Metabolism of thiopurine *S*-methyltransferases (TPMT). Metabolite: Azathioprine (AZA), Methylmercaptopurine (meMP), Methyl-thioguanosine monophosphate (meTGMP), Methyl-thioinosine monophosphate (mTIMP), Thioguanosine monophosphate (TGMP), Thioinosine monophosphate (TIMP), 6-mercaptopurine (6MP), 6-thioguanine (6TG). Enzyme: Glutathione-*S*-transferase A1, A2, M1 (GSTA1, GSTA2, GSTM1), Guanosine monophosphate synthetase (GMPS), Hypoxanthine guanine phosphoribosyl transferase (HPRT1), Inositol monophosphate dehydrogenase (IMPDH), Thiopurine methyltransferase (TPMT), Solute carrier family 8A2, 8A3, 29A1, 29A2 (SLC28A2, SLC28A3, SLC29A1, SLC29A2). Colors: blue—prodrug, drug or effect; green—inactive metabolites (adapted from [262]).

Pharmacogenomics of thiopurines (6-mercaptopurine (6MP) and 6-thioguanine (6TG)) with *TPMT* is probably the most studied drug/gene interaction in pediatric medicine. Thiopurines are applied as prodrugs that are converted into thioguanine nucleotides (TGNs) by hypoxanthine guanine phosphoribosyl transferase (HPRT) (Figure 2). TGNs are cytotoxic compounds that integrate into the DNA and RNA causing nucleic acid damage that ultimately leads to cancer cell death. TGNs can trigger apoptosis in normal immune cells causing serious ADRs mainly neutropenia, thrombocytopenia and hepatotoxicity manifested as veno-occlusive disease [95,106,111]. Inactivation of TGNs is carried out through *S*-methylation by cytosolic TPMT. Polymorphisms in the gene have been shown to exert an influence on TPMT activity [257,258]. For clinical purposes, individuals are divided into three major groups: normal, intermediate and poor metabolizers based on the presence of one or two loss of function alleles. Alleles *2, *3A,*3B and *3C are by far the most common variant alleles and are estimated to predict up to 90% of TPMT function and variability [259]. An additional 34 TPMT alleles have been described in different populations but with much lower frequencies [260].

This drug/gene association has important clinical implications because the treatment outcome of childhood ALL with 6MP is highly associated with maximum tolerable drug dose [111]. This is based on the observations of an inverse relationship between concentration of TGNs and *TPMT* genotype and the ability of the patients to tolerate full doses of 6MP. TPMT-poor metabolizers tolerated full 6MP dose only for 7% of the total weeks, in contrast to 65% and 84% for TPMT-intermediate and TPMT-normal metabolizers, respectively. TPMT-normal metabolizers missed only 2% of total treatment weeks, in contrast to 16% and 76% of missed weeks for TPMT-intermediate and TPMT-poor metabolizers, respectively [111]. Guidelines developed by CPIC recommend a normal dose for normal metabolizers. For intermediate metabolizers, a 30%–70% reduction is recommended for 6MP and a 30%–50% reduction for 6TG. Poor metabolizers receiving 6MP or 6TG should receive a 90% reduction in dose with drug administration three times per week in order to avoid ADRs (Table 2). Pre-emptive patient testing is highly recommended either to avoid adverse drug reactions in case of malignant disease or to reduce time needed for upward titration of drug dosage [256,261].

Recognizing the importance of ontogeny, CPIC reviewed the evidence on thiopurines/*TPMT* relationship gathered in pediatric cohorts and published a revised guideline in 2013. Because childhood leukemia is the most common childhood neoplastic disease, a large number of studies have been conducted in the pediatric population. However, evidence in children does not deviate from that seen in adults [255] and the revised CPIC guidelines do not recommend any specific adaptation for pediatric use [255].

#### 4.1.2. Thipurines/Nudix Hydrolase 15 (*NUDT15*) Pair

TPMT genotyping explains significant variability in response to treatment with thiopurines but substantial toxicities still occur in some patients with normal TPMT activity, suggesting other genetic predispositions must be involved in patient variability in thiopurine metabolism [123]. Recently, several replicating studies have shown that dose adjustment based on presence of low activity NUDT15 variant (Arg139Cys) should be made in children receiving 6MP for treatment of ALL [120,122,123,125,126]. In addition, the low activity allele was associated with alopecia and leukopenia in children treated with azathioprine [121,125,128,129] and myelosuppression in children treated for ALL with 6MP [120,128]. Moriyama et al. recently found three additional loss of function single nucleotide polymorphisms (SNPs) comprising at present four different loss of function alleles designated *2–*5 that were associated with thiopurine metabolism [123]. NUDT15 is a member of nudix hydrolase superfamily. It is homologous to nudix hydrolase 1 (MTH1) which is involved in the hydrolysis of 8-oxo-2′-deoxyguanosine-5′-triphosphate (8-oxo-dGTP), produced by oxidative damage, causing transversions due to DNA mispairing. Crystal structure and biochemical testing of NUDT15 have shown low catalytic activity towards deoxycytidine triphosphate (dCTP), deoxythymidine triphosphate (dTTP) and 8-oxo-dGTP but it has strongest activity against thiopurine derivatives of guanine (6-thio-dGTP). In addition, the same polymorphism associated with the above described clinical consequences have been shown to adversely affect NUDT15 activity. This suggests that a pool of normal guanine in cells having poor metabolizing variant of NUDT15 is affected more by thiopurines in comparison to cells carrying normal metabolizing *NUDT15* gene [263]. Although more evidence in terms of prospective clinical trials has to be gathered, *NUDT15* presents probably the most likely candidate for implementation of clinical guidelines because of its large effect and clinical significance especially in Asian populations with less frequent *TPMT* dysfunctional alleles. A recent study by Moriyama et al. suggests that, similarly with *TPMT*, *NUDT15* variants would likely benefit by tailoring dosing against the levels of thioguanosine triphosphate and thioguanosine-DNA giving a valuable direction for execution of further studies [123].

#### 4.1.3. Cisplatin/Xeroderma Pigmentosum, Complementation Group C (*XPC*) Pair

Cisplatin was the first platinum based chemotherapeutic used for the treatment of various types of cancers. It is, for example, highly effective for the treatment of neuroblastoma, osteosarcoma, hepatoblastoma and brain tumors predominantly found in children. Cisplatin acts as a DNA damaging agent through three different pathways: alkylating DNA, crosslinking guanines or inducing nucleotide mispairing. Because these events interfere with replication and transcription, cisplatin exerts cytotoxic function irrespective of the cell cycle. Cisplatin is mainly cleared through the kidney [264]. Although treatment with cisplatin has contributed greatly to overall survival of pediatric cancer patients its use comes at a cost [265]. The most severe ADRs in children are nephrotoxicity, neurotoxicity and ototoxicity [16,17]. A study looking at long-term effects of cisplatin found persistence of nephropathy even 10 years after treatment [266]. Ototoxicity in children is of particular concern because it can impair speech and normal development. A recent and ongoing clinical trial of Childhood liver tumors study group (SIOPEL-4) observed moderate or severe ototoxicity in 50% of children treated with cisplatin for high-risk hepatoblastoma [267]. Other studies in children with medulloblastoma, osteosarcoma and neuroblastoma detected permanent hearing damage in 60% of patients receiving cisplatin [268]. Based on the rationale that DNA repair mechanisms are involved in repairing damaged DNA caused by cisplatin, several studies explored the possibility of drug resistance, survival and toxicity association with particular DNA repair gene variations. Indeed, *XPC* gene of the nucleotide excision repair pathway (NER) was associated with ototoxicity in children and young adults treated for osteosarcoma [133]. An independent study showed the same *XPC* gene variant is associated with cisplatin induced neutropenia and hematological toxicity in adult patients treated for bladder cancer [134]. Even though evidence produced so far looks promising in terms of prediction of ADRs in patients receiving cisplatin, it must be emphasized that these two studies were performed on different patient populations with different treatment protocols. Further replication studies on a broader spectrum of cisplatin receiving patients are needed to verify the observed results before any guidelines can be established. The next question is related to the clinical utility of this particular association in light of inadequate replacement drugs or recommended dose intensity [269] and measures that can be taken to prevent ADRs. One option could be the refinement of cisplatin protocols to the levels that may minimize the ADRs or substituting with other platinum based chemotherapeutics like carboplatin that might produce less ADRs but still achieve the same efficacy.

### 4.2. Drugs with Moderate Pharmacogenetics Evidence

This section describes gene/drug associations with moderate strength of association according to PharmGKB. Table 1 summarizes drugs and their association with genes and variants, effect of variants, total number of publications, published in pediatric populations with details of the cohort analyses.

#### 4.2.1. Cisplatin and Carboplatin

Other moderate evidence genes associated with cisplatin are from the DNA damage repair pathways. Excision repair core complex (ERCC) 2, a helicase and a vital component of the NER pathway known to be involved in removal of DNA adducts formed after platinum treatment, was found to be useful for predicting clinical outcomes of osteosarcoma in children and young adults [133]. Other genes that present interesting targets for investigation in the context of pediatric patients are tumor protein 53 (*TP53*) [140,153], X-ray repair cross complementing 1 (*XRCC1*), *ERCC1* [140,142,144,155,157], methylenetetrahydrofolate reductase (*MTHFR*) [166,167] and *GSTM1* [148,149,150]. Their association with cisplatin and carboplatin is placed in level 2 evidence, however, no pediatric studies have been performed to date.

A pharmacogenetic study in children showed that hearing loss due to cisplatin treatment is associated with *TPMT* loss of function alleles [270]. Because of that, the Food and Drug Administration (FDA) had issued a special precaution on cisplatin drug labels stating that individuals with allele TPMT*3B and TPMT*3C (*3A is haplotype of *3B and *3C) are at a higher risk of hearing loss [270]. Despite the initial strong evidence between *TPMT* and cisplatin, PharmGKB qualified it as a level 3 drug/gene pair due to the lack of replication. In addition, two studies were unable to reproduce the association [271,272]; a third study was performed by the same group that reported the original association, this time with a smaller effect size [273], prompting the FDA to remove *TPMT* associated precautions from the cisplatin drug label.

#### 4.2.2. Methotrexate

**Figure 3:**
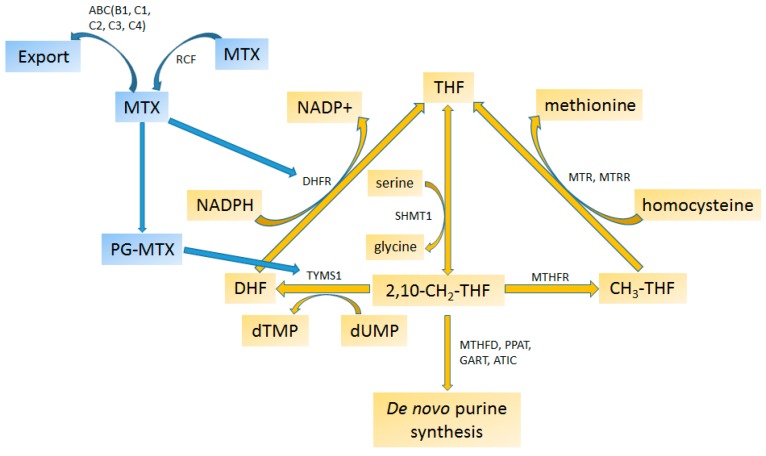
Functioning of Methotrexate. Metabolite**:** Dihydrofolate (DHF), Methotrexate (MTX), Methotrexate polyglutamate (PG-MTX), Methyl-tetra-hydrofolate (CH_3_-THF), Methylene-tetra-hydrofolate (2,10-CH_2_-THF), Nicotinamide adenine dinucleotide phosphate (NADP), Tetrahydrofolate (THF), Thymidine monophosphate (dTMP), Uridine monophosphate (dUMP). Enzyme**:** ATP binding cassette subfamily B1, C1, C2, C3, C4, (ABCB1, ABCC1, ABCC2, ABCC3, ABCC4,), Dihydrofolate reductase (DHFR), serine hydroxymethyltransferase 1 (SHMT1), Formyltetrahydrofolate synthetase (MTHFD), Methylenetetrahydrofolate reductase (MTHFR), Phosphoribosyl pyrophosphate amidotransferase (PPAT), Phosphoribosylglycinamide synthetase (GART), Replication factor C subunit 1 (RFC), Thymidylate synthase (TYMS1), 5-aminoimidazole-4-carboxamide ribonucleotide formyltransferase/IMP cyclohydrolase (ATIC), 5-methyltetrahydrofolate-homocysteine methyltransferase (MTR), 5-methyltetrahydrofolate-homocysteine methyltransferase reductase (MTRR). Colors: blue—active drug or metabolite; golden—endogenous metabolite (adapted from [22,207]).

Methotrexate (MTX) is a folate analogue that inhibits dihydrofolate reductase (DHFR), which converts dihydrofolate (DHF) to tetrahydrofolate (THF) needed for several one-carbon transfer reactions in nucleotide synthesis (Figure 3) [274].

MTX has been known for more than 60 years and was the first rationally designed molecule against cancer [275]. MTX remains an important anticancer agent, it is an essential component of consolidation and continuation therapy against ALL [276,277,278,279]. Despite the successful improvement of dosing regimens, there is still variability in therapeutic responses to MTX. There are a subset of patients who are more prone to the development of MTX-associated ADRs [274,278], whereas resistance to MTX remains a significant problem reducing the efficacy of the therapy. Resistance may arise through adaptation of cancer cells exposed to MTX, but can also be facilitated by genetic predispositions mainly involving variations in genes of folate metabolism, folate dependent enzymes and influx/efflux systems [274,278].

A most notable example of influx transporters is that of solute carrier organic anion transporter family member 1B1 (SLCO1B1 or OATP1B1) which was associated with high dose MTX clearance and toxicity [210,212,280]. Several SNPs in linkage disequilibrium (LD), including rs11045879, were in association, however, it is rs4149056 that was shown to have reduced MTX influx capacity [280]. A description of clinically important haplotypes in *SLCO1B1* is available at PharmGKB due to the involvement of the same gene in modulation of response to statins. This may be relevant for MTX use, especially for taking precautionary measures such as alkalization or increased hydration in carriers of dysfunctional *SLCO1B1* alleles. OATP1 expression levels are lower at birth and early childhood, but then slowly increase to reach adult levels after the age of 7 with genetic variants playing a major role in inter-individual variability in its expression at mature state [281,282]. ABCB1, another level 2 drug/gene pair, encodes a MTX exporter protein [168,169,170]. *ABCB1*-3435C>T was found to be associated with increased risk of neutropenia in Lebanese pediatric ALL patients [170] and with anemia and thrombocytopenia in a Danish population [168]. The other efflux transporters, ranked in a lower evidence group, like ABCC3 and ABCC4 also influenced the risk of relapse and ADR in childhood ALL [283,284]. Reduced folate carrier (RFC1) is responsible for MTX import. Even though at higher MTX doses uptake transporters might not play a major role, there are conflicting results on *RFC1* genetic variant and MTX response with higher incidence of relapse in individuals carrying defective *RFC1* variants [187,285,286].

Pharmacogenomic association studies intensively investigated genes of THF and MTX metabolism. MTHFR, responsible for conversion of 2,10-CH_2_-THF into CH_3_-THF and a main target of MTX, was most heavily investigated. Substantial evidence shows that *MTHFR* variants are associated with haematological toxicities [100,174], survival [180,190] and dose exposure [196] in children with ALL. However, two recent meta-analyses by Lopez-Lopez et al. and Hagleitner et al., concluded that variants of *MTHFR* were not good markers for toxicity and ADRs in pediatric ALL [179,186]. In contrast, a third meta-analysis was able to show positive association of C677T with relapse in childhood ALL [180]. The reason for discrepancies between meta-analysis and other studies are unknown but factors such as regiment protocols, patient populations and outcomes might obscure the effect otherwise presentable in a more homogeneous study group. 5-Methyltetrahydrofolate-homocysteine methyltransferase reductase (MTRR), involved in conversion of homocysteine to methionine was found to be associated with oral mucositis, increased platelet recovery [208] and increased thymidylate synthase (TYMS) catalytic activity [207] but not with decreased intelligence quotient (IQ) [185] in pediatric ALL patients.

Other genes from the THF metabolism, *DHFR*, *TYMS* and *SHMT1*, were assigned to a lower evidence group either because of contradictory reports or lack of replication in their association with ADRs, efficacy or pharmacokinetic. Variations in DHFR, a main MTX target converting DHF into THF, were shown to be associated with increased risk of relapse [287,288,289] and thrombocytopenia [190]. Another important enzyme, TYMS, that converts 2,10-CH_2_-THF to DHF, was found to be associated with relapse [290,291], stomatitis [286] or osteonecrosis [292] in children with ALL. There are however others that did not observe such association [187] or observe it only through interaction with other genes [177]. *TYMS* was also found to be associated with oral mucositis in patients receiving hematopoetic cell-transplantation [204]. Interestingly, increased catalytic activity of TYMS seems to correlate with *SHMT1* and *MTRR* variations [207]. Association studies on *SHMT1* were contradictory and few. One study found an association with liver toxicity in children receiving MTX treatment for ALL [208] while another did not detect the association with drug toxicity [177].

Even though metabolism of THF and MTX is well known, it is complex in terms of biochemistry and genetic variability. Ontogeny of these enzymes is not fully understood as shown, for example, by animal studies where variations in the expression of *MTHFR* genes of liver, kidneys and brain tissues during early foetal life and in neonates [293] were reported, suggesting that enzymatic phenotyping could add valuable data to genetic association studies. In addition, association studies used several different variations showing the need for harmonization by using the same variations in order to increase the power. One of the concerns in most of these studies is the definition of clinical outcomes, consideration of uniformity, and having more precision within the guidelines. Conducting explorative or replication studies with uniform design will enable evaluating the evidence in a systematic fashion with due consideration of multiple genes while controlling for confounders.

#### 4.2.3. Cyclophosphamide

**Figure 4:**
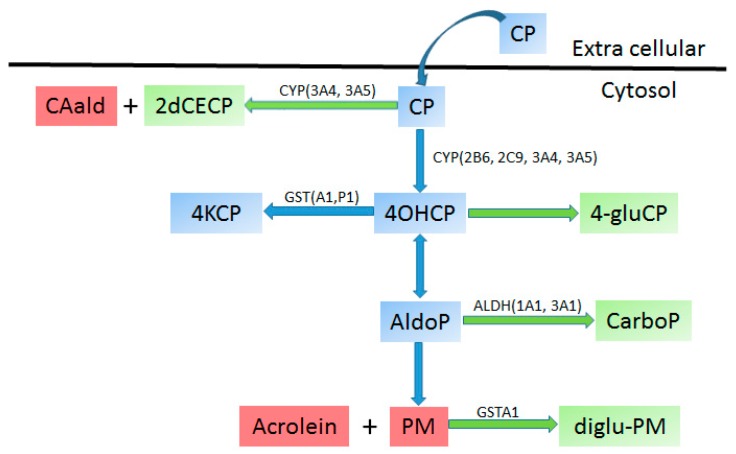
Metabolism of Cyclophophamide. Metabolites: Aldophoshphamide (AldoP), Chloroacetaldehyde (CAald), cyclophosphamide (CP), Carbophosphamide (CarboP), diglutathionylphosphoramide mustard (diglu-PM), Phosphoramide mustard (PM), 2-Dechloroethylcyclophosphamide (2dCECP), 4-Ketocyclophosphamide (4KCP). Enzymes: Cytochrome P450 3A4, 3A5, 2B6, 2C9 (CYP3A4, CYP3A5, CYP2B6, CYP2C9), Glutathione *S*-transferase alpha1, pi1 (GSTA1 GSTP1), Aldehyde dehydrogenase 1A1, 3A1 (ALDH1A1, ALDH3A1). Colors: blue—prodrug, drug; green—inactive metabolites, red—toxic metabolites (adapted from [22,299]).

Cyclophosphamide (CP) is used for treatment of various common [294,295] and rare childhood solid tumors such as osteosarcoma [296], Ewing sarcoma [296], rhabdomyosarcoma [297] and medulloblastoma [298]. CP is given as a prodrug that undergoes extensive metabolism before it is converted to the active form (Figure 4). Upon entering the system, around 70% to 80% of CP is converted to 4-hydroxycyclophosphamide (4OHCP) [299], a reaction catalyzed mainly by CYP2B6 and to a lesser extent by 2C9, 2C19, 3A4 and 3A5 [300,301,302]. 4OHCP tautomerizes into aldophosphamide that spontaneously changes to phosphamide mustard (PM) and acrolein [299]. It is PM that exhibits DNA crosslinking properties and ultimately causes cytotoxicity. Acrolein is water soluble and clears out of the system via urine, due to its high toxicity can cause haemorrhagic cystitis and other ADRs [303]. 4OHCP and PM are cleared by glutathione *S*-transferase (GST), mainly GSTA1 and GSTP1 [304,305]. Aldehyde dehydrogenase (ALDH) 1 and ALDH2 enzymes were shown to convert aldophosphamide to non-toxic carboxyphosphamide. CP is also converted directly to 2-dechloroethylcyclophosphamide. Although this reaction represents around 10% of total CP conversion, it is significant because it forms neurotoxic chloroacetaldehyde and is catalyzed by CYP3A4, CYP3A5 and CYP2B6 which is of concern when CP is used with other neurotoxic agents such as MTX or vincristine [302].

Current evidence for the involvement of CYPs, GSTs and ALDHs genetic variations in the pharmacokinetics of CP and 4OHCP is conflicting. A study by Erkhart et al. on 124 adults shows that variations in genes *CYP2B6*, *CYP2C9*, *CYP2C19*, *CYP3A4*, *CYP3A5*, *GSTA1*, *GSTP1*, *ALDH1A1* and *ALDH3A1* do not explain the inter-individual variability in CP and 4OHCP pharmacokinetics [306]. Others, in contrast, found an association of these pharmacokinetic parameters with variations in *CYP2B6* [307] and *CYP2C19* genes [308]. A recent study reported a link between CYP2B6*6 allele and reduced CP clearance in children with B-cell non-Hodgkin’s lymphoma [309]. Associations were also found between *MTHFR* and drug hematologic toxicity (aplasia, neutropenia, anemia and leukopenia) in children treated for osteosarcoma [188]. Other genes associated with cyclophosphamide efficacy and toxicity in adults include *GSTP1* [213,214], super oxide dismutase 2 (*SOD2*) [215] and *TP53* [151] but there is no report for these genes in children treated with CP. A particular problem when evaluating the genetic association of CP metabolism with CYPs, except for *CYP2B6*, is the ontogeny of CYPs in children at various developmental stages which changes the impact of variants. Additionally, CP is not used alone and most of the patients receive steroids at different doses, which are known to influence the expression of CYPs, and thus could alter the observations made in association studies. CP is used in different chemotherapy regimens at various dosing schedules and infusion times which can alter genotype–phenotype associations. The evidence reviewed here is of low to moderate and thus suggests that more comprehensive replication studies of CP are needed, especially in the field of pediatric oncology, before any clinical implementation would be possible.

#### 4.2.4. Irinotecan

**Figure 5:**
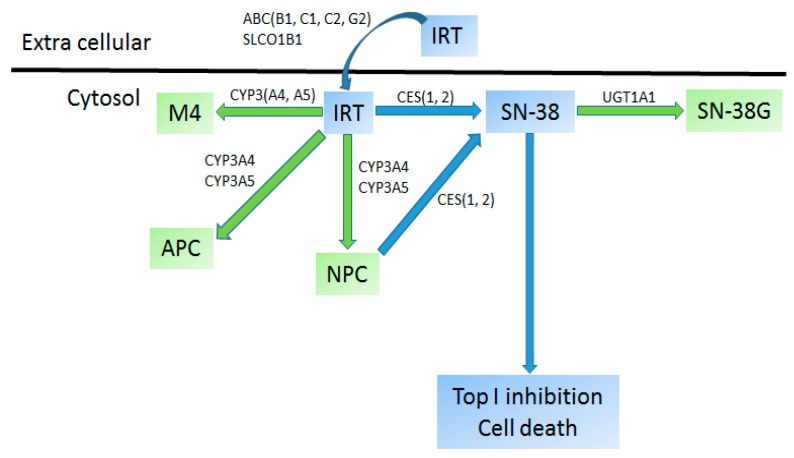
Metabolism of Irinotecan. Metabolites: Ethyl-10-[4-*N*-(5-aminopentanoic acid)-1-piperidino] carbonyloxycamptothecin (APC), Irinotecan (IRT), 7-ethyl-10-[4-(1-piperidino)-1-amino] carbonyloxycamptothecin (NPC), 7-Ethyl-10-hydroxy-camptothecin (SN-38), 7-Ethyl-10-hydroxy-camptothecin glucoronic acid (SN-38G), Irinotecan metabolite M4 (M4). Enzymes: ATP binding cassette subfamily B1, C1, C2, G2 (ABCB1, ABCC1, ABCC2 and ABCG2), carboxylesterase 1, 2 (CES1, CES2), Cytochrome P450 3A4, 3A5 (CYP3A4, CYP3A5), Solute carrier organic anion transporter family member 1B1 (SLCO1B1), UDP glucuronosyltransferase family 1 member A1 (UGT1A1). Colors: blue—prodrug, drug or effect; green—inactive metabolites (adapted from [22]).

Irinotecan (IRT) is most extensively used in the treatment of metastatic colorectal cancer but in pediatric medicine it is primarily used to treat patients with Rhabdomyosarcomas and Ewing sarcoma [310]. IRT is a prodrug that is spontaneously converted to the active compound 7-ethyl-10-hydroxy-camptothecin (SN-38) by carboxylesterase (CES) 1 and CES2 (Figure 5). It is SN-38 that inhibits topoisomerase 1 complex causing irreparable double strand breaks in DNA forcing cells to arrest in S phase and eventually undergo cell death. The major metabolic pathway for ITR (70%) involves uridine diphosphate (UDP) glucuronosyltransferase 1 (UGT1A1) enzyme that converts SN-38 into soluble and non-toxic SN-38 glucuronic acid excreted via the intestine. Other pathways for SN-38 clearance involve CYP3A4 and CYP3A5 oxidation that convert SN-38 into two inactive metabolites: APC (7-ethyl-10-[4-*N*-(5-aminopentanoic acid)-1-piperidino] carbonyloxycamptothecin) and NPC (7-ethyl-10-[4-(1-piperidino)-1-amino] carbonyloxycamptothecin), but the latter can be converted back to SN-38 by CES1 and CES2. Several transporters are involved in IRT transport: SLCO1B1, ABCB1, ABCC1, ABCC2 and ABCG2 [2,311]. Much like with other chemotherapeutics, overexposure of IRT can lead to ADRs such as myelosuppression, diarrhea and neutropenia [3,312]. Both UGT1A1 and CYP3A5 are well expressed in children and attain the adult level activity within 3–6 months post birth, hence genetic variants might predict the inter-individual variability to a large extent among children [313].

There are two variants with high frequency that both influence *UGT1A1* expression. UGT1A1*28 is a thymine-adenine (TA) repetition in the promoter region affecting the ability of RNA polymerase to initiate gene expression [314]. Another is UGT1A1*6 that causes G71 to R substitution but was shown to modulate UGT1A1 activity through gene expression [315]. Unlike UGT1A1*28, UGT1A1*6 is more common in East Asian populations where the frequency of UGT1A1*28 is lower in comparison to Europeans or African Americans [316]. The extent of compiled evidence mainly in adults treated for various kinds of cancers for association of IRT and *UGT1A1* persuaded several regulatory bodies including the FDA to issue a warning for UGT1A1*28 poor metabolizers. The same was also done by The Royal Dutch Pharmacists Association—Pharmacogenetics Working Group [261] and French Groupe de Pharmacologie Clinique Oncologique (GPCO-Unicancer) and French Réseau National de Pharmacogénétique Hospitalière (RNPGx) [316], recommending to use lower doses of IRT in UGT1A1*28 poor metabolizers in order to avoid ADRs. In pediatric populations, variations in *UGT1A1* showed no association with grade 3 and 4 neutropenia and diarrhea when used in low doses and in protracted protocol designed to avoid toxicity of irinotecan [249]. This is in agreement with a meta-analysis that reported association of *UGT1A1* genetic variants with irinotecan clearance in patients receiving higher or medium doses but not lower doses [230]. Studies in children with neuroblastoma explored the association between IRT-related ADRs and *UGT1A1*, suggesting that UGT1A1*28 poor metabolizers could be at risk of ADRs [317,318].

Two genes identified through a genome wide association study (GWAS) showing strong association with IRT ADRs were semaphorin (SEMA) 3C and chromosome 8 open reading frame 34 (C8orf34) [216]. SEMA3C is a member of semaphorin family known to promote survival and tumorigenicity possibly through angiogenesis [319,320,321] while the function of C8orf34 is unknown. Although these two genes might present interesting new associations, no replication studies exist to corroborate these results, therefore, further exploration of their clinical validity is needed.

#### 4.2.5. Vincristine

Vincristine is widely used as a combination chemotherapeutic agent for treating leukemias, lymphomas, brain tumors, neuroblastoma, Wilms tumor, rhabdomyosarcoma, Ewing sarcoma, and retinoblastoma in children [322]. Vincristine exert its cytotoxic effects by inhibiting microtubule formation thereby leading to mitotic arrest and cell death [323]. Approximately 25% of patients (both pediatric and adult) develop clinically significant vincristine-induced peripheral neuropathy (VIPN), necessitating the dose reduction or discontinuation of treatment [324]. CYP3A5 is predominantly involved in the metabolism of vincristine, and CYP3A5 is expressed only in 10%–20% of Caucasians (CYP3A5*1/*1) and more than 80% of African-Americans [325]. There are conflicting reports with majority of the studies favoring the association [324,326,327,328,329] of lower vincristine clearance in CYP3A5 poor metabolizers with higher risk for VIPN. There are also some population differences observed for this association [330].

A recent genome wide association study in a prospective cohort identified a variant in centrosomal protein 72 (*CEP72*) gene associated with VIPN during continuation phase of ALL treatment with a high number of vincristine doses [331]. In the same study, authors experimentally showed that sensitivity to vincristine was improved with lower expression of *CEP72*. These findings raised a hope for safe vincristine dose prediction in ALL treatment protocols. However, another retrospective study during initiation phase of ALL treatment in Spanish children could not replicate this association [332], owing to differences in the study design, population and the stage of the treatment protocol. At our hospital, we also observed severe neuropathy in CYP3A5 poor metabolizers independent of *CEP72* and *ABCB1* status [333]. The latter is a vincristine exporter protein, however, conflicting evidence of its association with treatment outcome in children with ALL exists [334,335,336,337].

Given that there is no difference in expression of *CYP3A5* between adults and children, this finding might have clinical value for predicting vincristine induced neuropathy in children. However, because the level of evidence is low, replication studies with the higher level of evidence are needed to implement genotype testing combining *CYP3A5*, *CEP72* and *ABCB1* for dose optimization of vincristine.

## 5. Conclusions

Upon reviewing the evidence gathered, sorted and published by PharmGKB (see Table 1), only the thiopurines/*TPMT* pair satisfies the highest standard of evidence needed for incorporation into clinical practice. Pediatric Pharmacogenomics recommendations for thiopurines do not differ from those used for adults. Particular attention should be paid to thiopurines/*NUDT15* association which has shown consistent results in multiple studies and has a large effect. Although the associated variant has low allele frequency in European populations, its frequency is almost 10% in East Asian populations (Table 1); resulting in a significant number of individuals with intermediate or poor metabolizing NUDT15 capacity. Cisplatin, the other drug in the group of strong evidence, was associated with *XPC*, but further association studies are clearly needed before the implementation into clinics. Moderate evidence gene–drug interactions were mostly produced for MTX pharmacogenetics. Studies are yet too few and hard to compare due to the variability across treatment protocols, diseases, populations and measurable outcomes. Irinotecan, on the other hand, has a strong association with *UGT1A1* to the extent that, for example, Dutch and French guidelines suggest reducing the dose by 30% in cases of UGT1A1*28 poor metabolizers and are scheduled to receive more than 250 mg/m^2^ [261,316]. Also, there is a possibility to use the lowest dose and protracted protocols in order to avoid ADRs. However, as illustrated in Table 1, there is a clear lack of data for pediatric cohorts suggesting that this gene/drug pair might be most promising for immediate research efforts. Even though metabolism of CY includes many well-known pharmacogenes, such as *CYP*s, *GST*s and *ALDH*s, association studies with pharmacokinetic produced conflicting data, thus showing the need to improve study designs to obtain conclusive results. Genes involved in oxidative stress response or genome stability affecting response to CY were classified as associations with moderate evidence, but have not yet been performed in children.

## 6. Future Directions

More systematic exploration of evidence based on prospective pediatric clinical trials is needed before any additional gene–drug interactions can be implemented in clinics. PharmGKB represents a valuable tool for clinicians and scientist for an overview and interpretation of the current data in pharmacogenomics. However, caution on interpretation of lower level gene–drug associations must be taken because the number of studies can vary substantially between gene–drug associations. A particular problem that the field of pediatric pharmacogenomic is faced with is ontology, which requires increased efforts to better understand the influence of genetic variants. Another problem facing future research is an increasing number of drug combinations used for treatment of cancer, which makes it harder to dissect the contribution of single therapeutic agents. A key for future research will be a better standardization of treatment regimens across multiple institutions and evaluation of genetic association in relation to complete treatment protocol.

## Figures and Tables

**Table 1:** Gene–drug pairs with high and moderate (Group 1 and 2) evidence of association as graded by PharmGKB.

Evidence Level	Drug	Gene	Allele/Variant	AFR	EAS	EUR	Effect	Total Articles	Pediatric Articles	All ref.	Ped. ref.	Condition (Pediatric)
Level 1	Thiopurines	*TPMT*	*2 rs1800462	0.001	0.000	0.006	Dosage, Toxicity/ADR	96	30	[23,24,25,26,27,28,29,30,31,32,33,34,35,36,37,38,39,40,41,42,43,44,45,46,47,48,49,50,51,52,53,54,55,56,57,58,59,60,61,62,63,64,65,66,67,68,69,70,71,72,73,74,75,76,77,78,79,80,81,82,83,84,85,86,87,88,89,90,91,92,93,94,95,96,97,98,99,100,101,102,103,104,105,106,107,108,109,110,111,112,113,114,115,116,117,118,119]	[89,90,91,92,93,94,95,96,97,98,99,100,101,102,103,104,105,106,107,108,109,110,111,112,113,114,115,116,117,118]	Acute lymphoblastic leukemia
			*3B rs1800460	0.003	0.000	0.028						
			*3C rs1142345	0.067	0.022	0.029						
			*4 rs1800584	NA	NA	NA						
		*NUDT15*	*2 and *3 rs116855232	0.008	0.095	0.002	Dosage, Toxicity/ADR	9	6	[120,121,122,123,124,125,126,127,128,129,130,131,132]	[120,121,122,123,124,125,126]	Acute lymphoblastic leukemia
	Cisplatin	XPC	rs2228001	0.249	0.333	0.405	Toxicity/ADR	2	1	[133,134]	[133]	Osteosarcoma
Level 2	Cisplatin	*ERCC1*	rs3212986 rs11615	0.291 0.037	0.299 0.262	0.250 0.622	Efficacy, Toxicity/ADR	11	0	[135,136,137,138,139,140,141,142,143,144,145,146,147]		NS
		*GSTM1*	Null	NA	NA	NA	Efficacy	3	0	[148,149,150]		NS
		*TP53*	rs1042522	0.669	0.414	0.285	Efficacy, Toxicity/ADR	5	0	[139,140,151,152,153]		NS
		*XRCC1*	rs25487	0.110	0.235	0.366	Toxicity/ADR	9	0	[136,137,138,139,140,154,155,156,157]		NS
	Carboplatin	*EGFR*	rs121434568	NA	NA	NA	Efficacy	8	0	[158,159,160,161,162,163,164,165]		NS
		*ERCC1*	rs11615	0.037	0.262	0.622	Efficacy, Toxicity/ADR	11	0	[135,136,137,138,139,140,141,142,143,144,145,146,147]		NS
		*MTHFR*	rs1801133	0.090	0.296	0.365	Efficacy	2	0	[166,167]		NS
		*XRCC1*	rs25487	0.110	0.235	0.366	Efficacy, Toxicity/ADR	9	0	[136,137,138,139,140,154,155,156,157]		NS
	Methotrexate	*ABCB1*	rs1045642	0.150	0.398	0.518	Toxicity/ADR	3	2	[168,169,170]	[168,170]	Lymphoma
		*ATIC*	rs4673993	0.095	0.294	0.313	Efficacy	2	0	[171,172]		NS
		*MTHFR*	rs1801133	0.090	0.295	0.365	Efficacy, Toxicity/ADR	37	26	[100,169,170,173,174,175,176,177,178,179,180,181,182,183,184,185,186,187,188,189,190,191,192,193,194,195,196,197,198,199,200,201,202,203,204,205,206]	[100,170,173,174,175,176,177,178,179,180,181,182,183,184,185,186,187,188,189,190,191,192,193,194,195,196]	Acute lymphoblastic leukemia
		*MTRR*	rs1801394	0.246	0.263	0.523	Toxicity/ADR, Metabolism/PK	3	3	[185,207,208]	[185,207,208]	Acute lymphoblastic leukemia
		*SLCO1B1*	rs11045879	0.189	0.453	0.190	Toxicity/ADR	4	3	[209,210,211,212]	[209,210,211]	Acute lymphoblastic leukemia
Level 2	Cyclophosphamide	*GSTP1*	rs1695	0.480	0.179	0.331	Efficacy, Toxicity/ADR	2	0	[213,214]		NS
		*MTHFR*	rs1801133	0.090	0.296	0.365	Toxicity/ADR	3	1	[151,188,204]	[188]	Osteosarcoma
		*SOD2*	rs4880	0.424	0.125	0.466	Efficacy	1	0	[215]		NS
		*TP53*	rs1042522	0.669	0.414	0.285	Efficacy, Toxicity/ADR	5	0	[139,140,151,152,153]		NS
	Irinotecan	*C8orf34*	rs1517114	0.424	0.122	0.363	Toxicity/ADR	1	0	[216]		NS
		*SEMA3C*	rs7779029	0.365	0.152	0.047	Toxicity/ADR	1	0	[216]		NS
		*UGT1A1*	rs8175347 rs4148323	NA 0.001	NA 0.138	NA 0.007	Toxicity/ADR	35	1	[217,218,219,220,221,222,223,224,225,226,227,228,229,230,231,232,233,234,235,236,237,238,239,240,241,242,243,244,245,246,247,248,249,250,251,252,253,254]	[249]	Solid tumors

Description of columns: 1: Evidence level; 2: drug name; 3: genes associated with drug; 4: genetic variant investigated; 5–7: Minor allele frequencies (MAF) were obtained from 1000 Genomes Consortium, Phase 3_V1-: AFR—African, EUR—European, EAS—East Asian; 8: Pharmacogenetic effect, 9: total number of articles; 10: number of pediatric articles; 11: references of total articles; 12: references of pediatric articles; 13: pediatric condition under investigation. NA—not available, NS—no study, PK—pharmacokinetic.

**Table 2:** Dosing guidelines for 6MP and TG based on the presence phenotype/genotype of TPMT.

TPMT Phenotype/Genotype	Dosing Recommendation 6MP	Dosing Recommendation 6TG
Normal metabolizer (two functional alleles)	Start with normal dose	Start with normal dose
Intermediate metabolizer (one functional allele)	Start with 30% to 70% reduced dose	Start with 30% to 50% reduced dose
Poor metabolizer (no functional alleles)	Start with 90% reduced dose, trice weekly	Start with 90% reduced dose, trice weekly

## Supplementary Materials

Supplementary materials can be found at www.mdpi.com/1422-0067/17/9/1502/s1.
