# Supplementary material for: Pharmacogenomics in Pediatric Oncology: Review of Gene—Drug Associations for Clinical Use
Source: Int J Mol Sci. 2016 Sep 8;17(9):1502. doi: 10.3390/ijms17091502 (PMC5037779; doi:10.3390/ijms17091502)
Supplement: Supplementary file 1 [file ijms-17-01502-s001.pdf]

Supplementary Materials: Pharmacogenomics in Pediatric Oncology: Review of Gene–Drug Associations for Clinical Use

Vid Mlakar, Patricia Huezo-Diaz Curtis, Chakradhara Rao Satyanarayana Uppugunduri, Maja Krajinovic and Marc Ansari

Table S1. Drug–gene pairs and level of evidence [1,22].

Agent	Pediatric Indication	Level 1	Level 2	Levels 3 and 4
6-Mercaptopurine	ALL/AML_APL	*TPMT*		*ABCB1; ABCC4; ADA; AOX1; CBS; COMT; CREB1; CYP3A4; CYP2D6; DPYD; GSTA1; GSTM1; GSTP1; GSTT1; G6PD; HLA-C; HPRT1; MTHFR; MTR; NNMT; NR3C1; NUDT15; ITPA; SLC19A1; SLC28A2; TPMT; TYMS; UGT1A1; UGT2B15; VDR; XDH*
6-Thioguanine	AML	*TPMT*		*ABCB1; ABCC4; ADA; AOX1; CBS; GSTA1; HPRT1; MTHFR; MTR; SLC28A2*
Bevacizumab	High-grade glioma			*CFH; DPYD; IL8; MTHFR; RGS5; SHMT1; VEGFA*
Bleomycin	Hodgkin lymphoma			*BLMH; CHRNB2; CNR1; GSTM1; GSTP1; GSTT1; ERCC2; FSHR; IL12B; NR3C1; PTGS2; SSTR2; TACR2; TNF; TNFRSF1B*
Carboplatin	Various brain malignancies		*MTHFR; EGFR; XRCC1; ERCC1*	*ABCB1; AKT1; ALDH1A1; ALDH3A1; ATP7A; BCL2; CYP1B1; DPYD; DSCAM; EIF3A; ERBB2; ERCC2; GGH; GSTA1; GSTM1; GSTP1; MAD1L1; MAP3K1; OPRM1; PTGS2; SLC19A1; SLC22A1; SLC22A2; SLC31A1; SLCA31A1; SLCA31A2; SOX10; TYMS; UGT1A1*
Carmustin	Various brain malignancies			*ABCB1; ABCC1; BLK; EPHX1; GSTA1; GSTM1; GSTP1; GSTT1; KCNH2; MGMT*
Cisplatin	Various solid malignancies	*XPC*	*TP53; XRCC1; ERCC1*	*ABCB1; ABCC3; ABCC4; ACYP2; ADH1C; AKT1; BAIAP3; COMT; CYP2E1; DCBLD1; EIF3A; EPHX1; ERCC2; ESR1; GALNT14; GSTA1; GSTA6P; GSTM1; GSTM3; GSTP1; GSTT1; KLC3; LIG3; LRP2; MAP3K1; MTHFR; MTR; MUTYH; NAT2; OTOS; RAD52; RPS20P2; SLC19A1; SLC22A2; SLC31A1; TPMT; UBE2I; VEGFA*
Cladribine	AML			*DCK; PDGFRA; PDGFRB; RRM1; RRM2; RRM2B; SLC29A3*
Cyclophosphamide	ALL and Hodgkin lymphoma		*MTHFR; GSTP1; NQO1; SOD2; TP53*	*ABCB1; ABCC1; ABCC4; ABCG2; ALDH1A1; ALDH2; ALDH3A1; CBR3; CRHR1; CRHR2; CYP1A2; CYP1B1; CYP2B6; CYP2C19; CYP2D6; CYP3A4; CYP3A5; EGFR; ERBB2; ERBB4; ERCC1; ERCC2; ESR1; ESR2; FOS; GSTA1; GSTM1; HTR3B; HTR3C; ICAM1; IL1B; IL1RN; IL4; IL6; IL10; IL12B; MAOA; MGMT; MMP3; MSH2; NOS3; NQO1; NQO2; PTGS2; SLC5A5; SLC22A16; TGFB1; TNF; TP53; VCAM1;VEGFA; XRCC1*
Cytarabine	ALL/AML			*ABCB1; ABCC1; ABCC6; CDA; CDK1; CYP2E1; CYP3A4; DCK; FCGR2A; GSTM1; GSTP1; GSTT1; MTHFR; NR3C1; NT5C3A; NT5E; SLCO1B1; SLC19A1; SLC22A12; SULT2B1; TIMP3; TPMT; TYMS; TP53; UGT1A1; VDR*
Dacarbazine	Hodgkin's disease, neuroblastoma			*CYP1A2; CYP2E3; GSTM1; IRF1; IL6; MGMT; MLH1; MSH2*
Dasatinib	CML			*ABCB1; ABCG2; ABL1; BCR-ABL; CYP1A2; CYP2C19; CYP2D6; CYP3A4; CYP3A5; EPHA2; FMO3; FYN; KIT; LCK; PDGFRB; SRC; UGT1A; YES1*

Table S1. *Cont.*

Agent	Pediatric Indication	Level 1	Level 2	Levels 3 and 4
Daunorubicin	ALL/AML			*ABCB1; ABCC1; CBR1; CBR3; CYP1B1; ABCB11; ABCC6; ABCG2; CYP1A1; CYP1A2; CYP1B1; CYP3A4; EGFR; GSTM1; GSTP1; GSTT1; MTHFR; NQO1; NOS3; NRP2; NR3C1; SLC19A1; SLC22A16; TAP1; TOP2A; TOP2B; TPMT; TYMS; UGT1A1*
Decitabine	AML			*CDKN2B; CDA; DCK; DNMT1; DPYD; EGFR; FOS; HBG1; ICAM1; MGMT; MMP2; MSH2; NOS3; NQO1; NTRK2; SLC28A1; TIMP3; TP53*
Docetaxel	Nasopharyngeal carcinoma			*ABCB1; ABCC1; ABCC2; ABCC6; ABCC8; ABCG2; ATP7A; BRCA1; BRCA2; CASP7; CHST3; CYP2C8; CYP2D6; CYP3A4; CYP3A5; CYP4B1; CYP19A1; DPYD; EPHX1; ERBB2; ERCC1; ERCC2; GFBR2; GSTMs; GSTP1; GSTTs; HNF4A; IGF2; IL1R2; IL1B; IL6; MAPK7; MDM4; MTHFR; NAT2; NR1I2; NDUFB4; OMR2; PIK3CA; PPARD; PPARG; PRDX4; PTGES; PTGS1; PTGS2; RPL13; SLC10A2; SLCO1B3; SPG7; SULT1C2; SULT1C4; TGFBR3; TNF; TPMT; TUBB; TYMS; TP53; UGT1A1; VEGFA; XPC; XRCC4*
Etoposide	ALL, AML and Hodgkin lymphoma			*ABCB1; ABCC1; ABCC2; ABCC3; ABCC6; ABCG2; ACSL3; ACSL5; AGPAT2; ALDH3A1; BRCA2; CYP1A2; CYP2A6; CYP2B6; CYP2C8; CYP2C9; CYP2E1; CYP3A4; CYP3A5; DYNC2H1; ERBB2; ERBB4; FOS; GSTA1; GSTM1; GSTP1; GSTT1; IL1B; IL6; UGT1A1; MTHFR; NQO1; NR3C1; PRNP; SLC19A1; TAP1; TNF; TOP2A; TOP2B; TOP2s; TPMT; TYMS; TP53; UGT1A3; VEGFA; VDR; WNT5B*
Everolimus	Subependymal astrocytoma			*ABCB1; BRAF; CYP3A4; CYP3A5; CYP2C8; HIF1A; KRAS; PIK3CA; VEGF*
Hydroxyurea	AML			*ABCB1; CCND1; CYP2D6; CYP3A4; HBB; KRAS; MAP3K5; MGMT; PDGFRA; SMN2*
Ifosfamide	For recurrence of Rhabdomyosarcoma			*ABCB1; ABCC4; ALDH1A1; ALDH2; ALDH3A1; CYP2A6; CYP2B6; CYP2C8; CYP2C9; CYP2C19; CYP2D6; CYP3A4; CYP3A5; GSTP1; GSTM1; GSTT1; IL1B; IL6; NR1I2; TNF*
Imatinib	ALL			*ABCB1; ABCC1; ABCC4; ABCC6; ABCG2; BCR-ABL; BCL2L11; BLK; CSF1R; CYP1A1; CYP1B1; CYP1A2; CYP2B6; CYP2C8; CYP2C9; CYP2C19; CYP2D6; CYP3A4; CYP3A5; CYP4FA; CYP4F3; DDR1; DDR2; FMO3; GSTT1; IFNG; IGF1; LCK; KIT; PDGFRA; PDGFRB; PIK3CA; SLCO1B3; IFNG; IGF1; LCK; PDGFRB; PIK3CA; RB1; RET; SLC22A1; SLC22A4; SLC22A5; SLCO1A2; UGT1A9; UGT2B15; VEGFA*
Irinotecan	Rhabdomyosarcoma		*UGT1A1; C8orf34; SEMA3C*	*ABCB1; ABCC2; ABCG2; CYP3A4; CYP3A5; SLCO1B1; UGT1A7; ABCC1; ABCC3; ABCC4; ACACA; AGT; ALDH1A1; APC; BCHE; BCR; CDK1; CDK6; CES1; CES2; CES3; CYP2B6; CYP1A2; CYP2C8; CYP2C19; CYP2D6; CYP2E1; DPYD; DRD5; EGFR; ERBB4; ERCC2; FOS; GSTP1; HLA-B; HLA-DRB1; IFNG; IGF1; IL1B; IL6; ITGA2; KCNQ5; MGMT; MLH1; MSH2; MT-ATP6; MTHFR; NQO1; NR3C1; PON1; PTGES; RB1; SLC19A1; TNF; TP53; TPMT; TYMS; UGT1A3; UGT1A4; UGT1A6; UGT1A9; UGT1A10; VEGFA; XRCC1; ACHE; CYP2A6; CYP2C9; CYP3A4; TOP1*
L-Asparaginase	ALL and Hodgkin lymphoma			*CYP3A4; GATA3; GSTM1; GSTP1; GSTT1; MTHFR; NR3C1; TYMS; UGT1A1*
Lomustine	Bone marrow suppression			*ABCB1; CYP2D6; CYP2D6; CYP3A4; MGMT*
Mechlorthamine	Hodgkin lymphoma			*ERBB2; IL8RB*

Table S1. *Cont.*

Agent	Pediatric Indication	Level 1	Level 2	Levels 3 and 4
Methotrexate	ALL/AML_APL		*MTHFR; SLCO1B1; ABCB1; ATIC; MTRR*	*ABCC1; ABCC2; ABCC3; ABCC4; ABCG2; ACOX1; ADA; ADORA2A; ALOX5; AOX1; ARID5B; CBS; CCND1; COMT; CREB1; CYP1A2; CYP3A4; CYP7A1; C18orf56; DHFR; DPYD; ENOSF1; ERBB2; FABP1; FMR1; GGH, GSTM1; GSTP1; GSTT1; G6PD; HLA-A; HLA-C; IL1B; IL1RN; IL6; ITPA; LIPC; MMP3; MTHFD1; MTR; NALCN; NAT2; NOS3; NQO1; NR3C1; PTPRM; RB1; SLC16A7; SLC19A1; SLC22A6; SLC22A7; SLC22A11; SLCO1A2; SHMT1; TGFB1; TNF; TNFAIP3; TNFRSF1B; TP53; TPMT; TYMS; UGT1A1; UGT2B15; VDR; XDH*
Nelarabine	T-cell ALL and T-cell Lymphoblastic lymphoma			*ADA*
Nilotinib	CML			*ABCB1; ABCC1; ABL1; ABCG2; BCR-ABL; CSF1R; CYP2B6; CYP2C8; CYP2C9; CYP2D6; CYP3A4; DDR1; DDR2; KIT; PDGFRA; SLC22A1; TYKs; UGT1A1*
Pazopanib	Rhabdomyosarcoma and Ewing tumor			*CYP3A4; HFE; FGFR1; FGFR3; FLT1; FLT4; ITK; KDR; KIT; LCK; PDGFRA; PDGFRB; UGT1A1*
Prednisone	ALL and Hodgkin lymphoma			*ABCB1; ABCC3; AGT; CYP2C19; CYP3A4; GSTM1; GSTP1; GSTT1; IL10; MTHFR; NOS3; NPPA; NR1I2; NR3C1; PDGFRA; SLC19A1; TGFB1; TYMS; UGT1A1; VDR*
Procarbazine	Hodgkin lymphoma			*MAOA; MGMT; MSH2; XDH*
Rituximab	Non-Hodkin lymphoma		*FCGR3A*	*FCGR2A; IL2; IL6; TGFB1*
Sunitinib	Gastrointestinal stromal tumors			*ABCB1; ABCC1; ABCG2; CSF1R; CYP3A4; KIT; FLT1; FLT3; FLT4; GFRAs; KDR; PDGFRA; PDGFRB; TYK2*
Temozolomide	Newly-diagnosed glioblastoma muliforme			*ABCB1; AGT; CYP3A4; MGMT; MSH2; PTGS2; TNF*
Temsirolimus	New treatment approaches for Rhabdomysarcoma			*ABCB1; CCND1; CYP2D6; CYP3A4; ESR1; FKBP1A; MTOR*
Teniposide	ALL			*ABCB1; ABCC3; CYP1A2; CYP2A6; CYP2B6; CYP2C8; CYP2C9; CYP2E1; CYP3A4; GSTM1; GSTT1; TOP2; TOP2A*
Thiotepa	Various solid malignancies			*ALDH1A1; ALDH3A1; CYP1A2; CYP2B6; CYP2C8; CYP2C9; CYP2C19;CYP2E1; CYP3A4; ERBB2; GSTA1; GSTM1; GSTP1; MGMT*
Topotecan	AML			*ABCB1; ABCC1; ABCC4; ABCG2; ACSL3; ALAD; AOX1; CAT; CCR5; CYP3A4; ERBB2; GCH1; LEPR; MLH1; MSH2; MTHFR; NR1I2; TAP1; TOP1; TOP1MT; TP53; VEGFA*
Vinblastine	Hodgkin lymphoma			*ABCB1; ABCB11; ABCC1; ABCC2; AOX1; BRCA2; CAT; CDK2; CYP2D6; CYP3A4; GGCX; GSTP1; IL1B; NR1I2; POR; SULT1A1; TP53; TUBA1B; TUBB; TUBB3*
Vincristine	ALL, Hodgkin's disease, non-Hodkin lymphoma, Wilms' tumor, neuroblastoma, rhabdomyosarcoma			*ABCB1; ABCB11; ABCC1; ABCC2; ABCC3; ABCC6; ACTG1; APOA1; CAPG; CAT; CEP72; CES1; CFTR; CYP3A4; CYP3A5; EGFR; GSTA1; GSTM1; GSTP1; GSTT1; IGF1; IL6; MAPT; MGMT; MTHFR; NR1I2; NR3C1; POR; PRNP; PTGS2; SLC19A1; STAT3; TNF; TP53; TPMT; TUBA1B; TUBB; TUBB3; TYMS; UGT1A1; VDR*

References

1. Cacabelos, R.; Fernandez-Novoa, L.; Carril, J.C.; Corzo, L.; Lombardi, V.; Tellado, I.; Carrera, I.; Bello, A.; Fraile, C.; Takeda, M. *World Guide for Drug Use and Pharmacogenomics*, 1st ed.; Euroespes Publishing: Corunna, Spain, 2012; p. 2920.
